# The effect of new e-commerce platform’s OSC promotion on consumer cognition: from cognitive legitimacy and cognitive psychology perspective

**DOI:** 10.3389/fnhum.2024.1380259

**Published:** 2024-05-30

**Authors:** Deng Yu, Han Wei, Zhang Xuefeng, Huang Zhongxuan, Zhang Yijun

**Affiliations:** School of Management, Southwest University of Political Science and Law, Chongqing, China

**Keywords:** e-commerce platform, online shopping carnival, cognitive legitimacy, cognitive process, event-related potentials

## Abstract

**Introduction:**

In the realm of emerging e-commerce platforms, the influence of online shopping events, specifically online carnival promotions (OSC), on consumer behavior is a significant area of interest.This paper delves into the effects of such promotions on consumer perceptions, a topic that has not been extensively explored in academic research.

**Methods:**

To investigate this phenomenon, two distinct studies were conducted. The first study employed a questionnaire-based experiment involving 220 participants, divided into two groups. The first study examined the mediating role of cognitive legitimacy in the relationship between OSC events organized by new e-commerce platforms and the perceptions of consumers. The second study utilized an event-related potentials (ERPs) experiment with 33 participants to explore the differences in consumer perceptions between OSC promotions and general promotions by new e-commerce platforms. This study measured the brain’s response to promotional stimuli to gain insights into the cognitive processes involved.

**Results:**

The first study yielded results that suggest OSC activities can facilitate the establishment of cognitive legitimacy for new e-commerce platforms. This, in turn, was found to be associated with an increase in positive purchase intentions among consumers. In the second study, the ERPs data indicated that exposure to OSC promotional materials elicited larger P2 and N2 ERP components when participants were presented with the logo of a new e-commerce platform. This was in contrast to the response to general promotional materials, suggesting a heightened cognitive and perceptual engagement with OSC promotions.

**Discussion:**

The findings from both studies collectively imply that OSC promotions have a distinct impact on consumer perceptions and cognitive processes. The implicit memory triggered by these promotions appears to influence the identification of new platforms and the mechanisms of cognitive control during online shopping. This, in turn, may have implications for explicit consumer behavior, suggesting that OSC promotions could be a powerful tool for shaping consumer attitudes and behaviors in the e-commerce space. The results underscore the importance of understanding the nuances of consumer engagement with new e-commerce platforms and the role of promotional strategies in fostering a positive brand image and consumer loyalty.

## 1 Introduction

The Online Shopping Carnival (OSC), akin to Alibaba’s “Double Eleven,” Jingdong’s “6.18” in China, or “Black Friday” in the United States, is widely recognized as a highly effective promotional campaign and branding festival within the retail industry. These carnivals are perceived as providing an alternative, unconventional experience characterized by eccentricity, suspension of hierarchies, and emotional engagement, distinct from everyday emotion (Adamo, 2008; Torn, 2012; O’Sullivan, 2016). In the realm of e-commerce, prominent retail platforms have effectively commercialized the carnival concept, generating a bustling shopping environment (Chen and Li, 2019) that often triggers herd behavior among consumers (Lennon et al., 2011). Nevertheless, the precise influence of either the reputation of major platforms or the popularity of the carnival events themselves on consumer attraction and purchasing intentions during the OSC remains a topic requiring further investigation.

In the context of emerging e-commerce platforms, the potential impact of Online Shopping Carnival (OSC) on consumer perception presents an intriguing area for research. Newly established platforms often face challenges associated with the “liability of newness” (Stinchcombe, 2000), which can hinder their legitimacy acquisition in the market legitimacy (Shepherd and Zacharakis, 2003). This issue is particularly pronounced in the mature online retail sector, as seen in China where newcomers struggle to establish themselves amidst dominant players like Alibaba, Jingdong, and Pinduoduo. Established platforms have effectively utilized OSC campaigns to attract consumers, suggesting that replicating such strategies could help new platforms overcome legitimacy obstacles (Warlop and Alba, 2004). Therefore, it is suggested that adopting OSC practices by new platforms may have a positive influence on consumer cognition processes.

Prior research has highlighted the positive influence of OSC promotions on well-established platforms such as the leading Chinese consumer-to-consumer (C2C) market player Alibaba (Liu et al., 2013; Schubert et al., 2022; Li et al., 2022b). However, the applicability of these findings to emerging platforms remains largely unexplored. The significance of renowned platforms in reinforcing C2C promotions is widely acknowledged (Swilley and Goldsmith, 2013; Zhao et al., 2019). While numerous studies have delved into the impact of OSC promotions on consumer behavior on established platforms (Lennon et al., 2011; Ibrahim and Wang, 2019; Shi et al., 2022), the effects of such promotions on consumers’ cognitive processes on new platforms are still uncertain. Furthermore, the influence of these promotions on the cognitive legitimacy ascribed to emerging platforms necessitates further investigation. Cognitive legitimacy pertains to consumers’ perceptions of a new venture’s credibility, distinct from socio-political legitimacy (Zimmerman and Zeitz, 2002; Delmar and Shane, 2004). For instance, the manner in which consumers comprehend a new enterprise and begin to perceive it as trustworthy shapes their assessment of the enterprise’s legitimacy (Aldrich and Fiol, 1994; Shepherd et al., 2000). While prior studies have predominantly focused on sales differentials and brand impacts resulting from OSC (Chen et al., 1998; Pauwels et al., 2002; Ataman et al., 2010), the cognitive distinctions and implications for the cognitive legitimacy of new platforms have not been thoroughly examined. This raises an intriguing inquiry: can an emerging e-commerce platform enhance its cognitive legitimacy by emulating successful carnival promotions from e-commerce giants like Alibaba or Jingdong, thereby enhancing consumer perceptions and heightening purchase intentions?

This paper presents two studies aimed at investigating the impact of the OSC promotion of a new e-commerce platform on consumer cognition. The first study examines the role of cognitive legitimacy as a mediator in the relationship between the platform’s OSC promotion and consumer behavior through a survey experiment. Existing literature suggests that OSC can generate excitement excitement (Qihua et al., 2020), interaction (Lam et al., 2001) and emotion connection (Aydinli et al., 2014), which attract consumer attention and engagement in promotional activities (Albuquerque et al., 2012; Chen et al., 2020), ultimately leading to increased interest in the platform or brand. Drawing on legitimacy theory, it is posited that increased interaction with the platform for information-seeking purposes can improve the platform’s evaluation, potentially resulting in enhanced traffic, reputation, and approval. The study proposes that new platforms can establish cognitive legitimacy and subsequently foster positive consumer behavior through OSC.

In the second study, we aim to investigate variations in consumer cognitive processes when presented with general and OSC promotions on a new platform, utilizing event-related potentials (ERPs) within a controlled laboratory environment. Modern neuroscience techniques such as functional magnetic resonance imaging (fMRI) and ERPs provide innovative perspectives on the correlation between cognitive functions and conventional marketing indicators (Dan and Gregory, 2010; Lee et al., 2017). By utilizing ERPs to capture cognitive processes (Picton, 1992; Geoffrey, 2004; Jonathan and Cyma, 2008), we can effectively evaluate consumers’ cognitive responses during OSC promotions, enhancing our theoretical framework. Our hypothesis suggests that consumers’ cognitive processes towards a new platform may exhibit notable distinctions between OSC and general promotions. Specifically, we anticipate variations in subjects’ attentional orientation, cognitive control, and cognitive categorization towards the new platform’s brand, which could be discerned through ERPs components.

This study aims to contribute significantly to the existing knowledge base by investigating the potential positive correlation between OSC promotions and consumer perceptions within the emerging platform context. Furthermore, the research seeks to assess the broader practical implications of carnivals and to shed light on the pivotal role of cognitive legitimacy as a link between carnival promotion and changes in consumer behavior. By examining the mediating function of legitimacy, this study takes an initial step towards clarifying the relationship between carnivals and consumer cognition in the realm of entrepreneurship. Additionally, the study integrates neuroscience and empirical research methodologies, including the use of ERPs and survey experiments in tandem. This comprehensive approach allows for a thorough evaluation of the effectiveness of OSC, pinpointing ERPs components that could serve as sensitive indicators. The study also introduces an innovative method for evaluating OSC through a combination of experimental questionnaires and ERPs.

## 2 Theory development and hypothesis

### 2.1 Online carnival promotion and consumers’ purchase intention

Drawing from the carnival theory, the excitement surrounding OSC promotions presents an unconventional and exhilarating option within a structured society (O’Sullivan, 2016). This concept is believed to provide a stark contrast to the monotony of daily life. Scholars in the field of communication have proposed that carnivals are characterized by exuberant yet irrational behaviors, often met with skepticism in traditional societal norms (Bakhtin, 1965/1984). Online shopping carnivals create a festive shopping environment that allows individuals to escape their ordinary routines and immerse themselves in a “second life” marked by the liberating and enthusiastic acquisition of products. Notably, OSCs are distinguished by three key elements: participatory engagement driven by attention, interaction centered around recommendations, and the emotional satisfaction derived from the experience, all of which have the potential to impact consumer perceptions and decision-making processes.

Firstly, OSC attracts consumers who actively monitor and participate in related events, exemplifying the promotion’s distinctive draw (O’Sullivan, 2016). Previous research has indicated that OSC commonly provides significant discounts (Lennon et al., 2011; Liu et al., 2021), which are especially attractive to cost-conscious consumers. These individuals are inclined to follow their preferred brands and engage with promotions on online shopping platforms, eagerly anticipating the most advantageous deals. This behavior persists even with lesser-known brands, as consumers actively seek out beneficial opportunities. Through their involvement in carnival activities, consumers become more familiar with the brands and products, with the knowledge acquired from these events influencing their purchase intentions.

Secondly, OSC is characterized by consumer engagements facilitated through recommendations. Prior to and during these events, new social connections are established, including brief interactions with strangers through comments (Zhang et al., 2020) and the exchange of deals among friends via messaging apps or online communities (Chen et al., 2021). These platforms actively promote communal participation to enhance the festive ambiance of the carnival. Additionally, emerging platforms utilize these promotional activities to provide avenues for consumers to share their thoughts, experiences, and interactions with others through recommendations, ratings, and presentations in exchange for coupons. When consumers receive recommendations, they may feel more assured in their purchasing decisions (Guo et al., 2016).

Thirdly, the enjoyment derived from OSC, which is typically unattainable in everyday life, is another distinguishing feature (Zhang, 2014; O’Sullivan, 2016). Consumers derive pleasure from the entertaining elements of carnivals, such as gaming features, shows, and giveaways, often sharing these experiences with their social circles. This enjoyment stems not only from the perceived value of the deals but also from the novelty of participating in these collective activities. Previous studies have shown that hedonic aspects of online shopping, such as perceived enjoyment, have a positive influence on consumer behaviour (Ziamou et al., 2012; Chiu et al., 2014). The positive emotions experienced during these carnivals can create a conducive mood for satisfaction with the shopping experience (Schau et al., 2009; Sirakaya-Turk et al., 2015).

In sum, OSC plays a crucial role in stimulating consumer engagement, fostering interactive communities, and generating enjoyment, all of which collectively contribute to increased purchase intentions. Consequently, we propose the following hypothesis:

*H1:* OSC promotion has a positive correlation with consumer purchase intention on new platforms.

### 2.2 Mediation of cognitive legitimacy

In the realm of emerging retail platforms and other new ventures without established market reputations, the risk of failure is notably high. Research in sociology often refers to the concept of the “liability of newness” as a key factor in business failures, emphasizing the importance of legitimacy (Choi and Shepherd, 2016). To address this risk, new ventures can work towards gaining legitimacy, typically through validation from external stakeholders (Singh; et al. Singh et al., 1986), which can then facilitate resource acquisition and customer engagement. In the context of new retail platforms, consumers are a critical stakeholder group from whom legitimacy must be obtained. Drawing on existing literature on consumer decision-making (Pollack et al., 2012; Maier et al., 2021; Kwak et al., 2022; Lortie et al., 2022; Li et al., 2022a), it is suggested that consumers’ perception of legitimacy may significantly influence the impact of a new platform’s promotional activities on consumer behavior.

From a consumer’s perspective, cognitive legitimacy is determined by the level of information dissemination about the new venture (Aldrich and Fiol, 1994). Enhancing cognitive legitimacy involves consumers gaining a deeper understanding of the products, services, and overall offerings of the new venture (Lortie et al., 2022). An intriguing question arises: can a new retail platform improve its cognitive legitimacy by implementing strategies similar to those used by established platforms, such as launching OSC? If affirmative, the new retail platform could consider adopting proven market tactics to establish legitimacy.

We argue that OSC can be an effective strategy for new platforms to establish cognitive legitimacy. By utilizing such campaigns, new platforms can gain recognition and comprehension among stakeholders, leading to increased stakeholder awareness and decreased uncertainty (Fisher et al., 2016). Implementing OSC enables the new platform to share organizational knowledge and information about the venture with consumers, thereby reducing uncertainty and strengthening legitimacy. Given that established platforms have successfully utilized similar promotional techniques (Lennon et al., 2011; Smith and Raymen, 2015), consumers are likely to acknowledge the new platform’s ability to conduct effective large-scale promotions and provide competitive pricing. Furthermore, a variety of promotional activities during events like carnivals help familiarize consumers with the platform’s offerings.

Consequently, cognitive legitimacy could serve as a mediator between OSC and consumers’ purchase intentions. By participating in carnival activities, consumers gather information and insights (Ailawadi et al., 2009; Chen et al., 2017), enhancing their understanding of the promotion and the platform itself (Burnkrant and Cousineau, 1975; Punj, 2011), which in turn reinforces the platform’s cognitive legitimacy. The process of acquiring information as described above informs consumers’ decision-making and impacts their purchase intentions (Shih et al., 2014). Additionally, the emotional connection established through carnival promotions creates a bond between consumers and the platform, further enhancing perceived legitimacy. This emotional involvement directs consumer attention towards the promotions and strengthens their inclination to make a purchase. Therefore, we put forward the following hypothesis:

*H2:* Cognitive legitimacy is mediated in the relationship of OSC promotion and consumers’ purchase intention

**Figure 1:**
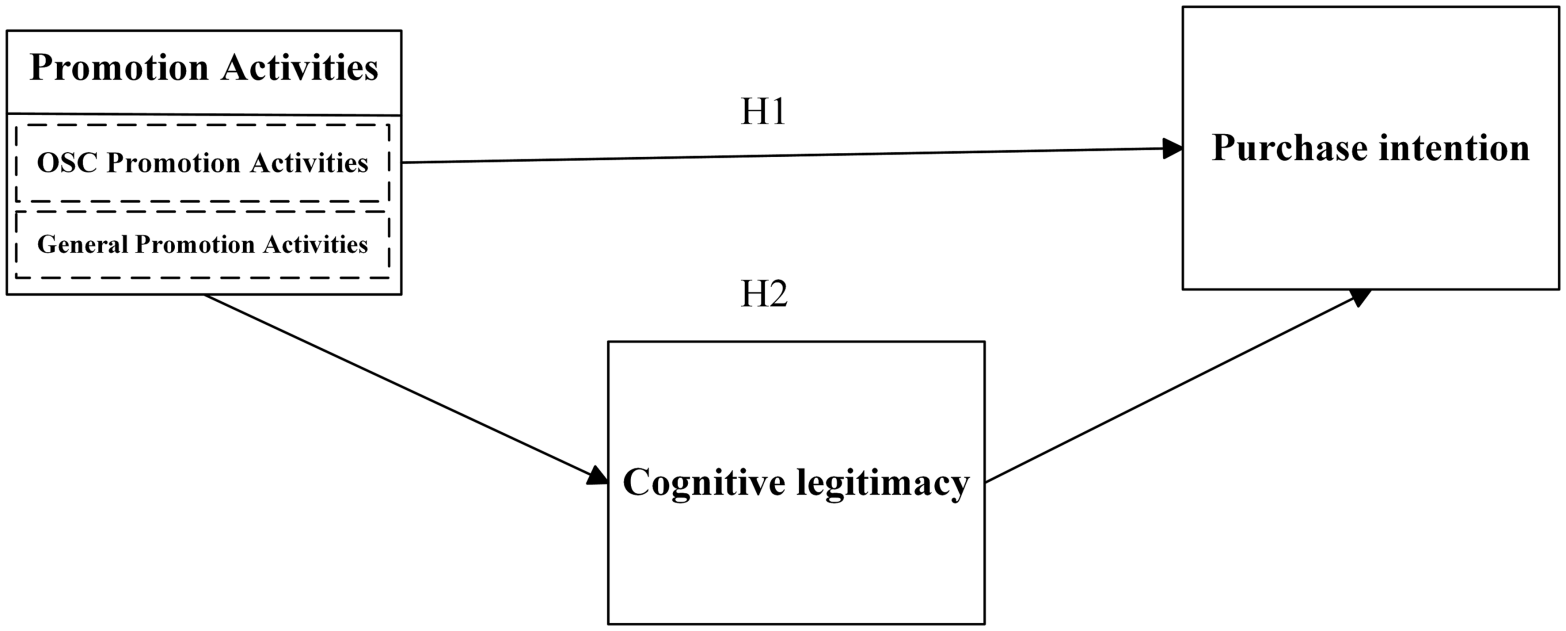
Conceptual framework of study.

The conceptual framework is shown in Figure 1.

## 3 Methodology

### 3.1 Study1

In study one, a questionnaire-based experiment was conducted to examine our hypothesis. The experiment involved two separate groups: the OSC promotion group and the general promotion group. Data gathering was carried out using WeChat, a prominent social networking platform in China. WeChat is a versatile application that allows users to communicate through instant messages, access current information, make payments, and share links. Participants initiated the survey by scanning a QR code on WeChat after accepting the invitation. Upon completion of the survey, respondents’ responses were promptly sent to the researchers.

#### 3.1.1 Participants

The survey data primarily originated from nine provinces in China, such as Chongqing, Zhejiang, Jiangsu, and Guangdong. Participants were randomly assigned to either the OSC promotion group or the general promotion group for the survey. Data collection took place in June 2022. Out of 235 responses collected, 220 were considered valid, resulting in an effective response rate of 93.6% after excluding 6 incomplete and 9 non-standard questionnaires, which were evenly distributed between the groups. The survey demographic included corporate employees, students, freelance entrepreneurs, small-business owners, and journalists. Participation was voluntary and uncompensated. The average age of participants was 26.09 years (SD = 5.05 years), with a gender distribution of 52.7% female and 47.3% male. On average, participants had been engaged in online shopping for 2.19 years (SD = 0.929 years), and around 68.56% had participated in promotional activities within the previous 6 months. The average monthly income of respondents was 3,056 RMB, with 12.3% earning more than 5,000 RMB per month.

#### 3.1.2 Experiment procedures and stimuli

**Figure 2:**
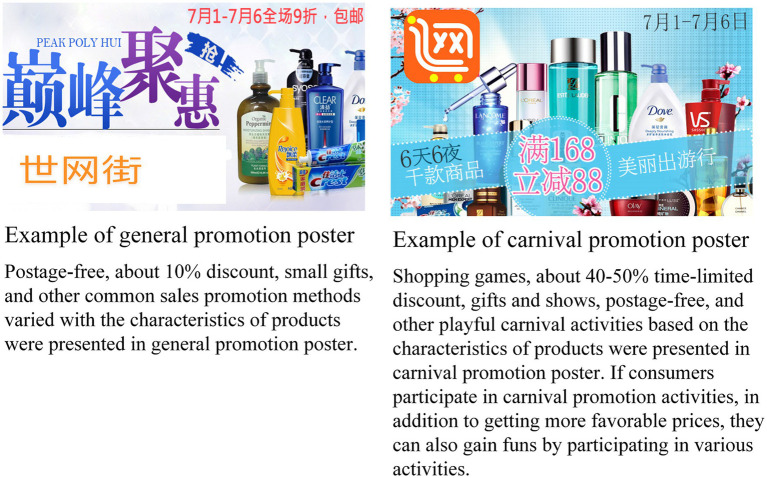
Examples of carnival and general promotion posters.

A nascent e-commerce platform in China was chosen as the subject of investigation to examine cognitive legitimacy and consumer purchase intentions following the introduction of OSC promotion activities. This platform operates on the emerging Factory to Consumer (F2C) model (Kauffman and Lee, 2010; Kumar et al., 2016), which directly connects manufacturers with consumers, eliminating various intermediary entities such as agents, distributors, and retailers. Unlike major market leaders like Alibaba and Jingdong, which hold significant market shares of 50.6 and 20.4% respectively, the platform chosen for this study had a market share of less than 0.1% according to the 2022 China e-commerce market data monitoring report by the China e-Business Research Center®. The selection of this nascent e-commerce platform was deliberate to avoid any biases associated with well-established platforms. The study introduced two distinct types of promotions: general market promotions and OSC promotions inspired by popular campaigns like “Double Eleven” on Alibaba and “618” on Jingdong. Sample posters for both types of promotions are depicted in Figure 2.

A preliminary examination was carried out to assess the distinctiveness and efficacy of two promotional strategies after the initial design of promotional posters. Feedback was sought from two experts in e-commerce to evaluate the rationale and complexities of the promotions, which resulted in enhancements to the visual presentation of the posters and the instructions for the questionnaire. Following this, 30 individuals knowledgeable in e-commerce were recruited to evaluate the revised carnival and general promotion posters, as well as the questionnaire guidelines. All participants concurred that the posters effectively represented the essence and purpose of the OSC promotions and general promotions, respectively. The survey was then distributed through WeChat, with participants randomly assigned to either the OSC or general promotion groups. All respondents completed identical sets of questionnaire items.

#### 3.1.3 Measurement

Drawing on existing literature, we constructed a survey instrument to evaluate the cognitive legitimacy of a recently launched e-commerce platform and its impact on consumers’ purchase intentions in the present study. The questionnaire items were derived from established scales developed by scholars both domestically and internationally, and were subsequently tailored to align with the nuances of the Chinese market. Prior to its formal dissemination, the questionnaire underwent a thorough evaluation by three experts to ensure its coherence, resulting in revisions that integrated their feedback as well as the outcomes of a preliminary test assessing its reliability and validity. Responses were captured using a 7-point Likert scale, where a rating of “1″ corresponded to “strongly disagree,” “4″ denoted “neutral,” and “7″ represented “strongly agree.”

The purchase intention was assessed through three items on a 7-point scale, including statements such as “The commodities of this e-commerce company’s promotion activities are what I need” (Liu and Zhang, 2014), “I think I will shop on the e-commerce platform in the future” (Foroudi et al., 2018), and “I think the commodities’ quality on the e-commerce platform can be guaranteed”(Foroudi et al., 2018). The mediator variable, cognitive legitimacy, was evaluated using a 4-item 7-point scale derived from existing cognitive legitimacy scales (Suchman, 1995; Goodrick and Reay, 2010; Pollack et al., 2012) and customized to suit the research objectives and e-commerce landscape in China. The items included affirmations like “I believe the e-commerce platform possesses strong economic capabilities,” “I am confident in the security of my money when shopping on the e-commerce platform,” “I think the e-commerce platform can enhance its market share through this promotional activity,” and “I have faith in the proficiency of the operators on the e-commerce platform.”

Control variables were selected based on the theory of consumer behavior (Lane, 1993; Grewal and Levy, 2007; Thaler, 2008) to examine their potential impact on consumer attitudes. Gender was categorized as “1” for male and “0” for female, income was evaluated through monthly earnings or living expenses, and online shopping experience was determined by the duration since the individual’s initial independent online purchase.

### 3.2 Study2

Event-related potentials (ERPs) serve as a more reliable analytical approach for investigating the primary effect in this study. ERPs are a fundamental tool in brain imaging research within the realm of cognitive neuroscience. This technology allows for the examination of consistent, internally generated electroencephalographic (EEG) activity triggered by both real and expected stimuli, whether they are visual or auditory. When combined with behavioral data, ERPs aid in exploring cognitive processes that are difficult to assess using traditional explicit evaluation methods. By utilizing ERPs, we aim to identify potential distinctions in consumer intentions when the newly established e-commerce platform launches an online OSC promotion versus a general promotion.

#### 3.2.1 Participants

Thirty-three participants (comprising 16 males and 17 females, with an average age of 21.8 ± 2.8 years) were recruited from the local community to partake in the research. All participants confirmed having normal or corrected eyesight and no prior history of neurological or psychiatric conditions, head trauma, or substance abuse. Furthermore, none of the participants were using any psychotropic medications during their involvement in the study. All participants were right-handed and local Chinese. Following approval from the local Medical Ethics Committee, each participant received a written informed consent document in compliance with the Declaration of Helsinki before the commencement of the experiment. A token of appreciation in the form of a small gift valued at around 5 US dollars was given to each participant upon completion of the experiment.

#### 3.2.2 Stimulus material

Informed by pertinent scholarly literature (Swilley and Goldsmith, 2013; Yu et al., 2018; Aldayel et al., 2021) and drawing inspiration from the notable success of large-scale promotional events like “Double Eleven” and Cyber Monday, two distinct sets of paired promotional posters were developed to accomplish this research. The differentiation between OSC and general promotional posters is primarily in the nature and extent of discounts offered, encompassing cash rebates, coupons, and time-limited sales, tailored to the specific attributes of the products. Reflecting the prevailing market conditions, OSC promotions feature more substantial discounts compared to general promotions. Furthermore, the carnival-themed posters incorporated interactive elements such as lucky draws, giveaways, and shows. Commencing promotions within a week following the conclusion of the experiment was strategically chosen to bolster participant engagement.

**Figure 3:**
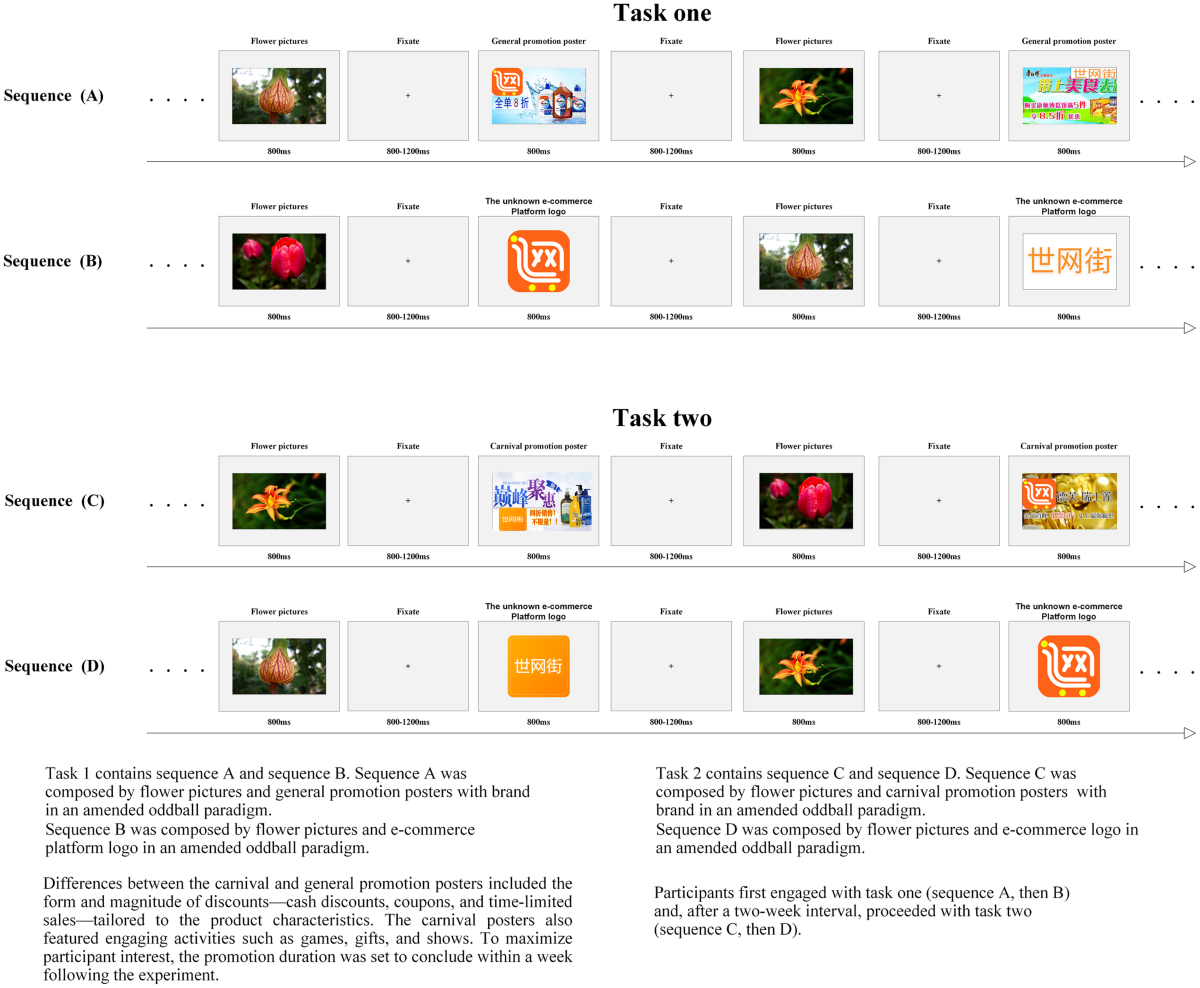
Schematic drawing of the paradigm.

Figure 3 illustrates the primary types of stimuli utilized in the study, along with their respective timings. The stimuli consisted of three vividly colored floral images, five general promotional posters featuring the nascent platform logo, five OSC promotional posters also showcasing the nascent platform logo, and five variations of the platform logo. To enhance participant familiarity with the items featured in the promotional posters, the study categorized the items into four groups: daily essentials, snacks, clothing, and electronics. The subsequent debriefing session revealed that while participants recognized the products in the posters, they were not familiar with the e-commerce platform and had not engaged in any transactions on it.

#### 3.2.3 Experimental procedure

The study consisted of two tasks, as illustrated in Figure 3. Task one included sequences A (comprising three floral images and five general promotion posters) and B (consisting of three floral images and three platform logo pictures). Task two involved sequences C (comprising three floral images and five OSC promotion posters) and D (matching sequence B). The platform logo was consistently placed in the corner of the promotion posters. A modified oddball task was utilized, with the three floral images acting as target stimuli across all sequences. The standard stimuli were general promotion posters or OSC promotion posters in sequences A and C, respectively. In sequences B and D, the standard stimuli were the nascent e-commerce platform logo pictures. To minimize carry-over effects between sequences, the experiment was split into two sessions. Participants first completed task one (sequence A, then B) and, after a two-week break, proceeded with task two (sequence C, then D).

The study was carried out in a shielded environment free from electromagnetic and acoustic disturbances to reduce external influences. Participants were seated comfortably and observed an experimental series displayed on an LCD screen using E-Prime software (version 2.0). The stimuli were presented in a pseudo-randomized order, with each stimulus appearing more than 40 times. Each trial began with a fixation cross, followed by the presentation of stimuli. The size of the stimuli was adjusted to guarantee clear visibility for the participants. Participants were asked to identify the number of floral images in each sequence and verbally communicate their responses to the researcher. Participants with data accuracy below 95% were excluded from the analysis. A preliminary practice session was conducted before the main experiment to familiarize the tasks and ensure task adaptation.

## 4 Results

### 4.1 Data analysis – study 1

The tools employed in this research were derived from established scales that had been validated by scholars in China and globally, demonstrating strong reliability and validity. Statistical analysis was conducted using SPSS version 21.0, which verified the robustness of the scales: all Kaiser-Meyer-Olkin (KMO) values surpassed 0.719, factor loadings ranged from 0.520 to 0.765, and Cronbach’s alpha coefficients for each construct exceeded 0.809, indicating excellent internal consistency.

#### 4.1.1 Descriptive statistics and correlations

In this study, the data analysis involved conducting descriptive and correlation analyses on the dependent, independent, mediator, and control variables using SPSS version 21.0. The results, as presented in Table 1, indicated statistically significant positive correlations. Specifically, there was a strong positive correlation between carnival promotion and cognitive legitimacy (r = 0.536, *p* < 0.001), as well as a positive correlation between carnival promotion and consumers’ purchase intention (*r* = 0.141, *p* < 0.05). Additionally, a notable positive correlation was observed between consumers’ purchase intention and cognitive legitimacy (r = 0.634, p < 0.001), and no indications of multicollinearity were found.

**Table 1:** Descriptive statistics and correlations.

Variables	Mean	SD	1	2	3	4	5	6
Consumers’ purchase intention	4.040	0.977	1					
Carnival promotion	0.529	0.500	0.141^*^	1				
Cognitive legitimacy	4.663	1.085	0.634^***^	0.536^***^	1			
Gender	1.480	0.501	−0.021	0.151^*^	0.082	1		
Age	3.318	1.070	−0.011	−0.038	0.026	0.184^**^	1	
Shopping time	4.1031	1.12432	0.004	0.095	−0.036	−0.016	−0.084	1

#### 4.1.2 Results

In this study, hierarchical regression analysis was utilized to investigate the influence of OSC promotions on consumers’ purchase intentions. The regression coefficients are detailed in Table 2. The initial model (Model 1) included only control variables, while Model 2 assessed the impact of carnival promotions on purchase intention.

**Table 2:** Result of analysis of regression and the effects mediating of cognitive legitimacy.

Variables	M1	M2	Path	Beta	LLCI	ULCI
Gender	−0.039	−0.086	***Direct effect***			
Age	−0.006	0.002	Carnival promotion→ Cognitive legitimacy → Consumer intention	−0.5569	−0.7884	−0.3254
Shopping time	0.003	−0.009				
Carnival promotion		0.290*	***Indirect effect***			
∆R^2^	−0.013	0.004^*^	Carnival promotion→ Cognitive legitimacy → Consumer intention	0.8464	0.6119	1.0932
R^2^	0.001	0.022				
F	0.038	1.21				

^+^Indicates significance at the *p* ≤ 0.1 (*p ≤ 0.05, **p ≤ 0.01, ***p ≤ 0.001) level of confidence.

H1 proposed a positive relationship between carnival promotions and purchase intention. The results from Model 2 supported this hypothesis, with carnival promotion emerging as a significant predictor at a significance level of 0.05 (*β* = 0.290). This suggests that carnival promotions have a positive impact on consumers’ purchase intentions.

In order to investigate the mediating role of cognitive legitimacy in the association between carnival promotion and consumers’ purchase intentions, a bootstrap regression analysis was conducted. Following the principles of bootstrap methodology, Model 4 was employed to analyze the mediating effect of cognitive legitimacy. The coefficients representing the mediation effect are presented in Table 2.

The results of the bootstrap analysis revealed a statistically significant indirect effect (Index = 0.8464), with the 95% confidence interval not excluding zero (LLCI = 0.6119, ULCI = 1.0932). Additionally, the direct effect was also found to be significant (Index = −0.5569), with its 95% confidence interval excluding zero (LLCI = −0.7884, ULCI = −0.3254). These findings suggest that cognitive legitimacy partially mediates the relationship between carnival promotion and consumers’ purchase intentions, thereby providing support for Hypothesis 2.

### 4.2 Data analysis – study 2

#### 4.2.1 Electroencephalogram recording and analysis

Participants wore a 32-channel EEG cap (Quickcap, Neuroscan, Victoria, Australia) while performing tasks, with electrodes positioned based on the international 10/20 system. The left mastoid was used as the reference electrode, and the ground electrode was positioned between FpZ and Fz. Horizontal and vertical EOG signals were also recorded and later removed during offline data processing. Electrode impedance was kept below 5 KΩ during recording. EEG data for sequences B and D were collected using the Neuroscan® EEG system (Neurosoft Labs Inc., Victoria, Australia), with a band-pass filter ranging from 0.01 to 100 Hz and a sampling rate of 500 Hz. The acquired data were processed offline using Curry 7.0 SBA (Neurosoft Labs Inc.). Any significant artifacts caused by eye or muscle movements were manually eliminated, and epochs with EOG amplitudes exceeding 200 μV, or showing signs of amplifier saturation or baseline shifts over 250 μV/s, were excluded, resulting in a 7% data rejection rate. The onset time of the nascent e-commerce platform logo was used as the reference for segmenting ERPs, covering a period from −200 ms to 800 ms (pre-stimulus to post-stimulus). Mean ERP amplitudes, triggered by the nascent e-commerce platform logo in sequences B and D following promotion, were calculated and compared using repeated-measures ANOVA.

#### 4.2.2 Results

**Figure 4:**
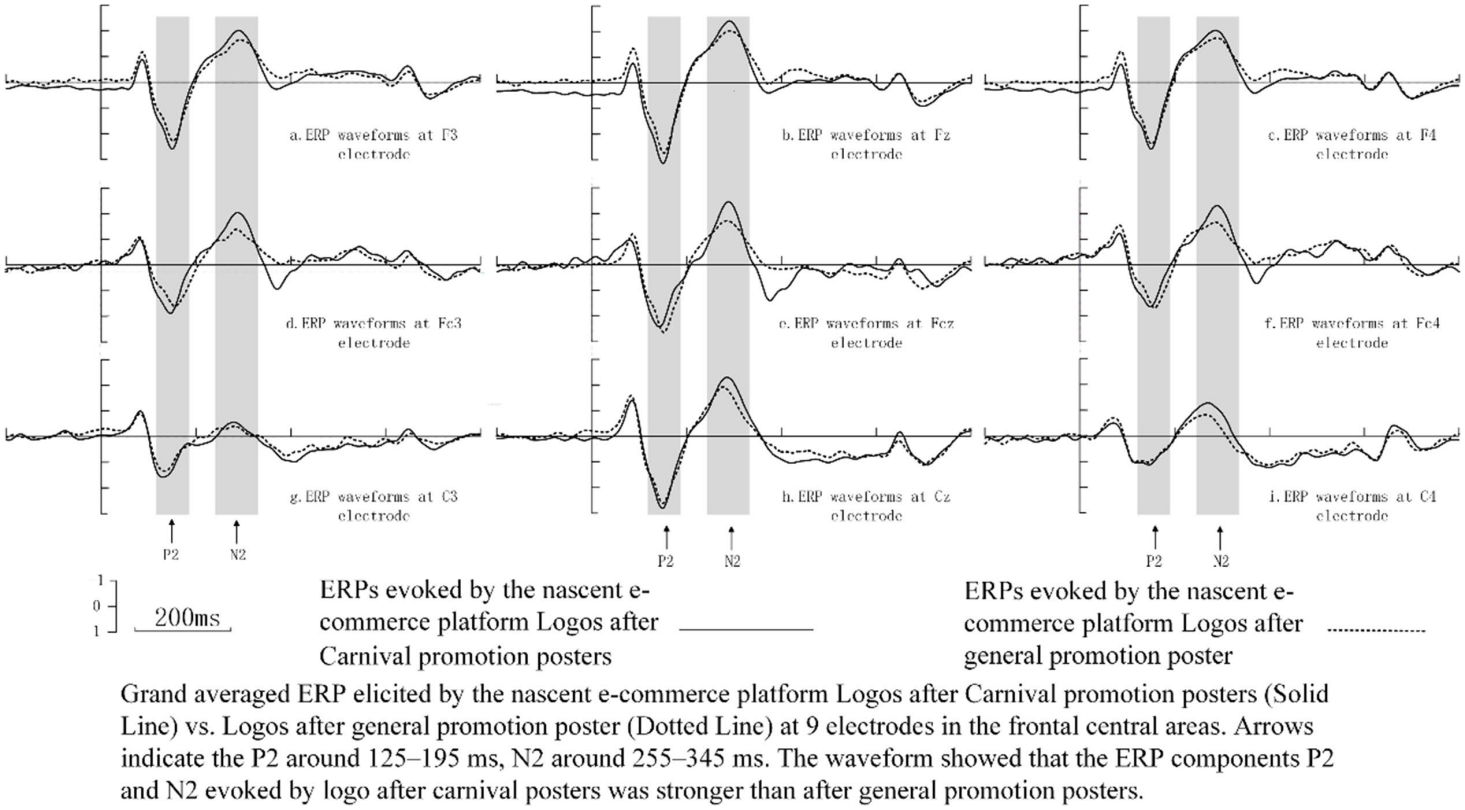
Raw ERPs waveforms at 9 electrode sites.

**Figure 5:**
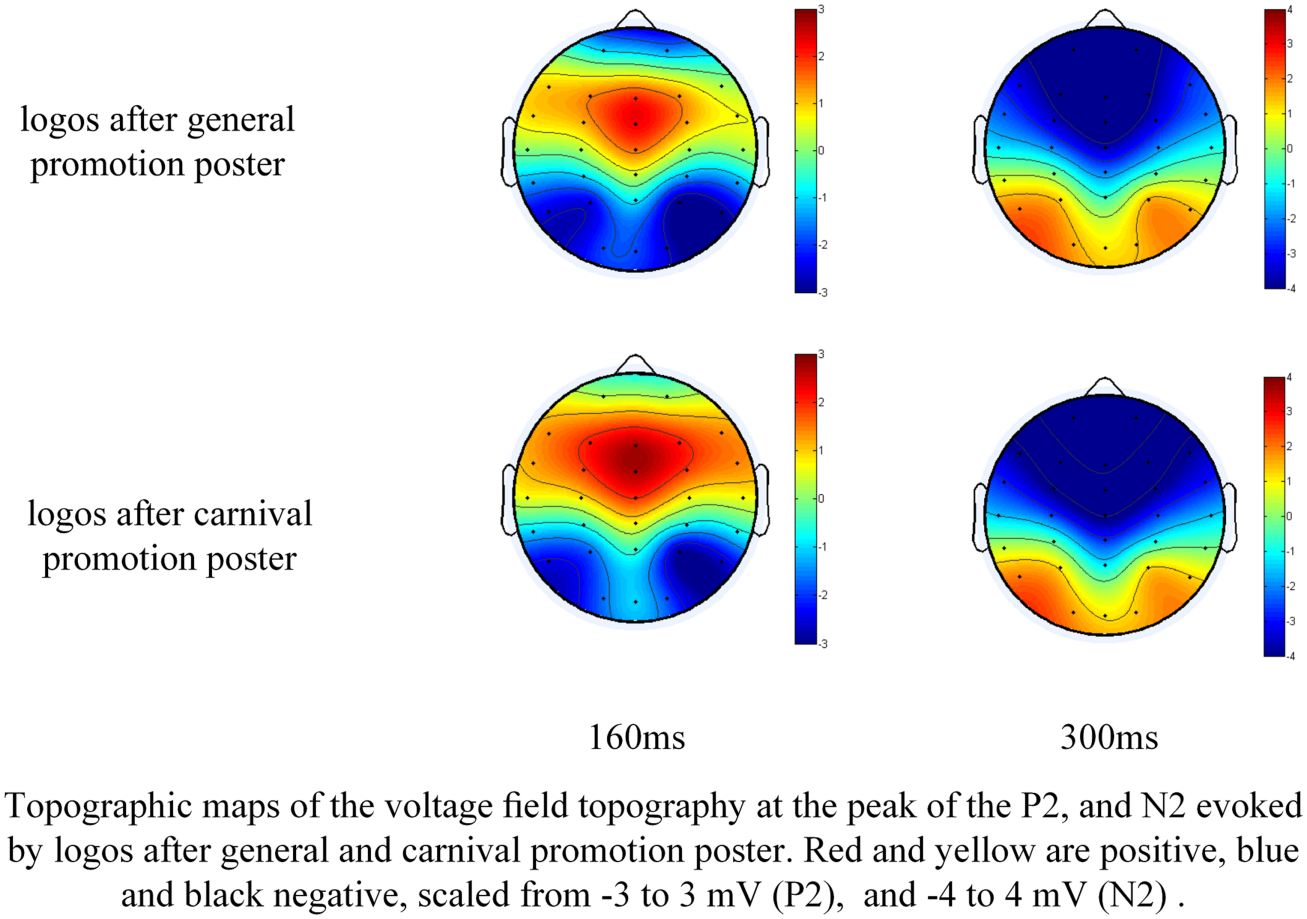
Topographic maps of the voltage field topography.

Figure 4 illustrates the ERP components elicited by the nascent e-commerce platform logo in frontal and central regions. Nine electrode sites (F3, Fz, F4, FC3, FCz, FC4, C3, Cz, and C4) were selected for statistical analysis based on visual assessments of potential distributions, topographical maps (see Figure 5), and previous studies (Geoffrey et al., 1996; Anika et al., 2013; Fu et al., 2022). The average amplitudes of the P2 (125-195ms) and N2 (255–345 ms) components were assessed through a repeated-measures ANOVA to investigate neural reactions to the platform logo following general and OSC promotions at the aforementioned electrode sites. The ANOVA factored in the type of stimulus (general versus OSC promotion) and the electrode site (F3, Fz, F4, FC3, FCz, FC4, C3, Cz, and C4). Greenhouse–Geisser corrections were applied as necessary, and contrasts were examined with a significance level set at *p* < 0.05. Table 3 provided descriptive statistics for the P2 and N2 components elicited by the nascent e-commerce platform logo after general promotion and OSC promotion at the specified nine electrodes.

**Table 3:** Basic descriptive statistics of evoked potentials.

Distribution		After general promotion	After OSC promotion	*t* value	*p* value
P2	F3	1.67 ± 0.78	2.38 ± 1.26	−2.328	0.016
	Fz	2.21 ± 1.21	2.66 ± 1.26	−2.595	0.027
	F4	1.54 ± 0.84	1.98 ± 0.88	−4.362	0.003
	FC3	1.73 ± 0.53	1.98 ± 0.53	−2.258	0.028
	FCz	2.33 ± 0.62	2.81 ± 0.81	−2.593	0.019
	FC4	1.26 ± 0.61	1.57 ± 0.79	−3.125	0.009
	C3	1.17 ± 0.59	1.36 ± 0.63	−3.357	0.003
	Cz	1.72 ± 0.67	2.12 ± 0.74	−2.689	0.014
	C4	0.26 ± 0.43	0.53 ± 0.62	−2.652	0.043
N2	F3	−2.33 ± 0.83	−2.88 ± 1.47	2.253	0.016
	Fz	−2.65 ± 0.72	−3.69 ± 1.32	2.692	0.025
	F4	−2.37 ± 0.65	−3.33 ± 1.41	2.564	0.018
	FC3	−1.53 ± 0.69	−2.39 ± 1.26	2.635	0.017
	FCz	−2.29 ± 0.67	−3.29 ± 1.22	2.258	0.016
	FC4	−1.56 ± 0.82	−2.02 ± 1.03	2.962	0.010
	C3	−0.38 ± 0.65	−0.69 ± 0.58	2.657	0.003
	Cz	−1.59 ± 0.49	−1.97 ± 0.74	3.548	0.003
	C4	−0.46 ± 0.78	−0.84 ± 0.68	3.568	0.002

The mean amplitude of P2 and N2 (mean ± SD) recorded at nine electrodes in time windows of 125–195 ms, 255–345 ms, and two-sample paired t-tests for different sites after general or OSC promotion poster.

The P2 component exhibited a significant main effect [*F* (1, 32) = 42.198, *p* < 0.001] attributed to the promotion condition, indicating that the average amplitudes of P2 elicited by nascent e-commerce logos under the OSC promotion condition surpassed those under the general promotion condition. Additionally, the distribution displayed a significant main effect [*F* (8, 256) = 49.892, *p* < 0.001], with higher mean amplitudes predominantly observed across midline electrodes. A significant interaction effect between promotion and distribution was also noted [*F* (8, 256) = 45.687, *p* < 0.001]. Further examination revealed a more positive P2 component in response to OSC promotions compared to general promotions, as evidenced by raw waveforms and scalp topographical mapping. This enhancement was particularly prominent in the frontal-central scalp region, peaking at the center of the frontal-central area.

Regarding N2, the findings indicated a significant impact of the promotional condition [*F* (1, 32) = 17.356, *p* < 0.001], where the average amplitudes following the promotion of the nascent platform logo post-OSC were lower compared to those subsequent to general promotion. Moreover, a significant influence on amplitude distribution [*F* (8, 256) = 115.659, *p* < 0.001] was identified, with mean amplitudes being notably reduced along the midline compared to other electrode locations. Additionally, a significant interplay between promotion and distribution [*F* (8, 256) = 12.254, *p* = 0.003] was observed. The nascent platform logo following the OSC promotion elicited a more distinct negative N2, particularly evident in the frontal-central regions of the scalp.

## 5 Discussion

In China, online shopping on platforms such as Alibaba and JD.com has become a well-established behavior. The shopping carnivals hosted by Alibaba or JD.com attract a massive population to their platforms every year (Zhang, 2014; O’Sullivan, 2016; Yan et al., 2016; Liu et al., 2021; QuestMobile, 2022; XinhuaNet, 2022).It can be inferred that Alibaba and JD.com have gained significant recognition from key stakeholders—the public—and have achieved a high level of cognitive legitimacy. However, the new e-commerce platform chosen for this study has a very low market share in China, and the participants surveyed were not familiar with the platform. For this platform to survive and develop, it must adopt various means to obtain cognitive legitimacy from stakeholders.

From this perspective, cognitive legitimacy has been considered as a mediator between the carnival and consumer purchase intention in this study. According to Baron and Kenny’s method for determining mediator variables (Baron and Kenny, 1986), to test for the existence of a mediating effect, one must first assess the significance of the path relationship between the independent variable and the dependent variable. It is necessary to check whether the independent variable is significantly related to the dependent variable. On the premise that the independent variable is significantly related to the dependent variable, if both direct and indirect effects exist simultaneously, the mediator plays a partial mediating role; if the indirect effect exists and the direct effect does not, the mediator plays a complete mediating role. The results show that there is a significant positive correlation between the carnival promotion and consumer purchase intention (r = 0.141, *p* < 0.05), and cognitive legitimacy has both a direct effect (Index = −0.5569) and an indirect effect (Index = 0.8464) on the relationship between the carnival promotion and consumer purchase intention, indicating that cognitive legitimacy plays a partial mediating role in this study.

The introduction of the study has already pointed out that there are essential differences between carnival promotions and general promotions in terms of the way the activities are conducted and the enjoyment people derive from participating in them. In a market context where consumers are accustomed to frequent general promotions, even if the organizer of a shopping carnival promotion similar to that of Alibaba or JD.com is the new e-commerce platform adopting the F2C model selected for this study, the pull effect of the shopping carnival can significantly enhance consumer purchase intention (*r* = 0.141, *p* < 0.05). However, whether it can truly promote consumer purchase intention depends on the level of cognitive legitimacy perception consumers have towards the new e-commerce platform (as evidenced by the partial mediating role of cognitive legitimacy in the relationship between the carnival promotion and consumer purchase intention, Index = −0.5569). That is to say, consumers might see that the new e-commerce platform has launched a carnival-style promotional event but may still not trust the platform, meaning that occasional carnival promotions may not have broken down the cognitive legitimacy barrier for consumers towards the new e-commerce platform.

The reason for this outcome is that the three aspects of legitimacy—cognitive legitimacy, normative legitimacy, and regulatory legitimacy—play different roles at different stages of the corporate lifecycle, such as the founding phase, growth phase, and maturity phase (Yang et al., 2015; Fisher et al., 2017; Islam et al., 2018). Among them, cognitive legitimacy mainly plays a role in the early stages of the company (Pollack et al., 2012; Li et al., 2014; Almobaireek et al., 2016). The e-commerce platform selected in this study adopts a more aggressive F2C model, allowing products to go directly from the factory to the consumer, which is significantly different from the C2C and B2C models familiar to consumers (the main e-commerce models adopted by Alibaba and JD.com are C2C and B2C models). In fact, this e-commerce company is challenging the traditional norms of consumers and the e-commerce market. In this case, although carnival promotions can play a stimulating role in enhancing consumer purchase intention, they may still not fully convince consumers to trust the e-commerce company. After all, occasional carnival promotions may not fully convince consumers that the company has strong economic strength, or that shopping on this e-commerce platform is as safe in terms of financial security as on Alibaba or JD.com, or that consumers believe the e-commerce platform has excellent operators. Therefore, we believe that in this study, the enhancement of cognitive legitimacy for this e-commerce company may mainly come from gaining more market share through this promotional activity. We further believe that if the company can convey other aspects, such as further demonstrating strong economic strength, a well-established financial security system, or the excellent background of the operators, it can further enhance the recognition from key stakeholders and improve consumers’ perception of the company’s legitimacy, ultimately bringing substantial benefits to the enterprise.

From the perspective of consumer psychology, after the new e-commerce company held a carnival promotion activity that mimicked Alibaba and JD.com, there was indeed a change in the cognitive process of the participants towards the new e-commerce company. This change can be inferred from the P2 and N2 components evoked by the company’s logo after the participants were exposed to the carnival promotion advertisement and the general promotional stimulus advertisement.

The P2 component, which typically emerges approximately 150–200 ms after stimulus presentation, is primarily influenced by the task relevance of stimuli (Debora et al., 2009) and is commonly referred to as the anterior P2 (Geoffrey et al., 1996; Erika et al., 2013). This component, predominantly observed in front-central regions, is associated with the process of identifying targets (Geoffrey, 2004; Erika et al., 2013). In this study, the unfamiliar e-commerce platform logo were designated as standard stimuli in sequences B and D. Participants were initially exposed to either general or OSC promotion posters in sequences A or C. Subsequently, they were tasked with identifying the target stimuli—flower pictures—within these sequences, as well as the nascent platform logo. The increased P2 amplitude following exposure to OSC promotions, in comparison to exposure to general promotions, indicates more robust processing and heightened attention allocation to the nascent platform’s logo stimuli. This finding suggests that OSC promotions were likely to have a lasting impact on participants, even when the promoter was an unfamiliar platform and the standard stimuli were intentionally disregarded, in line with common expectations.

An alternative hypothesis posits that the P2 component’s target sensitivity could enhance the top-down facilitation of object recognition (Kopp et al., 2007). The P2 component is believed to reflect this sensitivity during the visual identification of objects. In Sequences A and C, the logo of the nascent e-commerce platform was consistently positioned in the corner, and participants were not explicitly instructed to focus on the logo during the study; nevertheless, the logo was still visually processed and transmitted from the visual cortex to the prefrontal cortex throughout the experiment (Victor and Pieter, 2000). Participants may demonstrate increased receptiveness to information conveyed through OSC promotion posters compared to general promotion posters, possibly due to the former implying greater cost savings. As a result, the event-related potentials (ERPs) of the P2 component triggered by stimuli associated with the emerging platform were intensified following the presentation of OSC promotion posters.

The findings indicated that logos featured in OSC promotion posters elicited a stronger N2 response compared to logos in general promotion posters, suggesting that implicit memory plays a role in shaping neural responses to logo stimuli in in sequences B and D, even in the absence of conscious recognition or retrieval of the logos from the promotions (Daniel, 1987). The initial phase of the experimental task revealed that differences between OSC and general posters led to the encoding of logo information at varying levels of implicit memory in the participants’ brains (Larry et al., 1987; Bruce and Abhijit, 2002). Implicit memory associated with OSC promotions demonstrated greater resilience compared to that from general promotions, supporting previous claims about the malleability of implicit memory (Arnel, 1998; Kathmann et al., 2006). During the second phase of the experiment, participants, while engaged in counting flower pictures, had their cognitive control processes influenced by the strength of implicit memory related to the platform’s logo. In sequences B and D, participants had to suppress the influence of less relevant stimuli (logos) to focus on the primary task of identifying flower pictures, requiring the allocation of additional cognitive resources to inhibit the logo’s implicit memory and prioritize the primary task for controlled behavior. Consequently, a more negative N2 response was observed for logos following OSC promotions compared to those following general promotions. An alternative explanation suggests that the N2 response may reflect attentional processes, such as the detection of novel stimuli and the direction of visual attention within the visual cortex.

## 6 Findings and contributions

### 6.1 Findings

The purpose of this research is to evaluate the impact of online promotional strategies on consumer purchase intentions through two separate studies. In study 1, we observed that an OSC promotion can lead to increased purchase intentions among consumers for a nascent platform. This effect is partially attributed to the platform establishing cognitive legitimacy through the particular promotion activities. Consequently, we have elucidated the connection between OSC promotion and consumer intention by considering cognitive legitimacy as a key factor.

In study 2, we investigated the cognitive discrepancies experienced by consumers when engaging with an unfamiliar emerging platform after exposure to two distinct online shopping promotions. The P2 and N2 components, triggered by the nascent e-commerce platform, were more pronounced following OSC promotions in comparison to general promotions. These results indicate that early cognitive responses to a new platform, as indicated by P2 and N2 components, are influenced by the nature of the promotion, suggesting alterations in target identification and cognitive control mechanisms. While OSC promotions led to higher P2 and N2 amplitudes than general promotions, subsequent classification processes may not be solely impacted by promotional content. Our findings suggest that event-related potential (ERP) components could serve as valuable indicators for assessing the effectiveness of promotional tactics, even in cases where the new platform is unfamiliar to participants.

### 6.2 Contributions

The study presented in this research paper contributes significantly to the existing literature in several aspects. Firstly, it represents one of the earliest examinations of the impact of OSC promotions on newly established platforms and their influence on consumer purchase intentions. By demonstrating the positive correlation of carnival promotions on consumer intent, the study offers novel insights into the utilization of such promotions within the context of emerging platforms. Previous research has predominantly focused on the effects of these promotions on well-established platforms, such as Alibaba’s “Double Eleven,” where the outcomes are often intertwined with the platform’s reputation (Swilley and Goldsmith, 2013; Schubert et al., 2022). This study expands the understanding of the relationship between carnival promotions on nascent platforms and consumer purchase intentions, emphasizing the importance of considering the promotional context in relation to consumer intent.

Secondly, the research advances the comprehension of consumer cognitive processes that are impacted by online carnival promotions, moving beyond a mere examination of behaviors. While prior studies have concentrated on the characteristics of carnival promotions to develop theoretical frameworks (Punj, 2011; Li et al., 2022b) or have explored their effects on consumer behavior (Lennon et al., 2011; Chen and Li, 2019), the cognitive variations induced by carnival promotions specifically on new platforms have received limited attention. Through the utilization of empirical data and analysis of ERP components, this study provides robust evidence of the distinct effects of carnival promotions on consumer intentions.

Lastly, the research delves into the role of cognitive legitimacy in the connection between carnival promotion and consumer intention, a dimension often overlooked in studies that solely focus on direct behavioral influences. The findings suggest that new platforms can enhance perceived cognitive legitimacy through carnival promotions, consequently boosting consumer purchase intentions. While existing literature predominantly views carnivals as marketing tactics (O’Sullivan, 2016) or promotional strategies that may trigger herd behaviors or other forms of social influence (Lennon et al., 2011; Zhao et al., 2019; Liu et al., 2021). This study proposes that carnivals can also serve as a viable method for new platforms to establish legitimacy. This insight sheds light on the operational mechanism of carnival promotions in the entrepreneurial sphere and underscores their significance for new platforms seeking to overcome the challenges associated with being new entrants in the market.

### 6.3 Implications and future research

In order to minimize the potential influence of well-established e-commerce giants on our research findings, we opted to focus on an emerging platform in China for our investigation. The results indicate that promotions related to online shopping carnivals have a distinct impact on early cognitive processes compared to general promotions. Consequently, businesses seeking to effectively attract consumer attention may find value in implementing artificial online shopping carnivals as a viable strategy.

Online shopping carnivals differ from conventional promotions in various aspects, encompassing not only significant discounts but also a variety of additional promotional activities. Drawing inspiration from successful events such as “Double Eleven” or Cyber Monday, leading e-commerce entities in different markets could potentially introduce similar carnival-style promotions for mutual benefit.

Furthermore, this study demonstrates that alterations in P2 and N2 components, which signify target recognition and cognitive control adjustments, in response to different promotions can be objectively and quantitatively monitored using event-related potentials (ERPs). ERPs not only supplement conventional research methodologies but also enable the examination of pre-behavioral judgment, real-time monitoring, and post-behavioral prediction and evaluation of consumers’ cognitive processes. ERPs offer a pathway for understanding the cognitive mechanisms that underlie consumer behavior through the lens of cognitive neuroscience, providing a neuroscientific framework for comprehending consumer behavior.

Future research endeavors could broaden the scope to include offline consumption contexts. Exploring whether high-value durable goods like new luxury items or local services could benefit from carnival-style promotional strategies would be of interest. Additionally, employing more sophisticated experimental designs to better replicate real-world promotional settings and to dissect consumer cognition with greater precision is recommended. From a technological perspective, forthcoming studies should leverage the unique capabilities of ERP technology and explore its integration with eye-tracking, fMRI, and other physiological measurement tools. This integrated approach would facilitate a more comprehensive exploration of the cognitive mechanisms that influence consumer behavior.

## Data availability statement

The raw data supporting the conclusions of this article will be made available by the authors, without undue reservation.

## Ethics statement

The studies involving humans were approved by the Southwest University of Political Science and Law Ethics Committee. The studies were conducted in accordance with the local legislation and institutional requirements. Written informed consent for participation in this study was provided by the participants’ legal guardians/next of kin.

## Author contributions

DY: Writing – review & editing, Supervision, Project administration. HW: Writing – review & editing, Validation, Funding acquisition. ZX: Writing – original draft, Conceptualization. HZ: Writing – review & editing, Software, Data curation. ZY: Writing – review & editing, Software, Data curation.
